# 环磷酰胺单药治疗T细胞大颗粒淋巴细胞白血病疗效及停药后疗效维持情况分析

**DOI:** 10.3760/cma.j.cn121090-20241024-00416

**Published:** 2025-07

**Authors:** 乐乐 张, 林珠 田, 虹 潘, 珍 高, 伟望 李, 若难 李, 婧余 赵, 金波 黄, 馨 赵, 建平 李, 能 聂, 潇 于, 丽云 李, 哲湘 匡, 力维 方, 均 施

**Affiliations:** 1 中国医学科学院血液病医院（中国医学科学院血液学研究所），血液与健康全国重点实验室，国家血液系统疾病临床医学研究中心，细胞生态海河实验室，天津 300020 State Key Laboratory of Experimental Hematology, National Clinical Research Center for Blood Diseases, Haihe Laboratory of Cell Ecosystem, Institute of Hematology & Blood Diseases Hospital, Chinese Academy of Medical Sciences & Peking Union Medical College, Tianjin 300020, China; 2 天津医学健康研究院，天津 301600 Tianjin Institutes of Health Science, Tianjin 301600, China

**Keywords:** 白血病，大颗粒淋巴细胞, 环磷酰胺, 无治疗缓解, Leukemia, large granular lymphocytic, Cyclophosphamide, Treatment free remission

## Abstract

**目的:**

探索环磷酰胺单药治疗T细胞大颗粒淋巴细胞白血病（T-LGLL）的疗效及停药后疗效维持情况。

**方法:**

收集2019年6月至2024年3月中国医学科学院血液病医院再生医学诊疗中心收治的37例接受环磷酰胺单药口服治疗的T-LGLL患者临床资料，分析其临床特点、疗效及长期无治疗缓解（TFR）情况。

**结果:**

37例T-LGLL患者中位年龄为60（37～86）岁，男22例（59.5％）。30例（81.1％）表现为贫血，28例（75.7％）符合继发性纯红细胞再生障碍标准，15例（40.5％）出现粒细胞减少或缺乏，11例（29.7％）存在淋巴细胞计数增多，3例（8.1％）血小板减少。既往未接受过免疫抑制治疗的16例（43.2％）患者为初治组；免疫抑制治疗复发或无效的21例（56.8％）患者为复发/难治组。所有患者符合治疗指征，接受环磷酰胺（50～100）mg/d口服，可评价疗效的36例患者中达血液学缓解25例（69.4％），中位达血液学缓解时间为2.0（0.7～7.0）个月；初治组与复发/难治组血液学缓解率差异无统计学意义（68.5％对66.7％，*P*＝0.589）。25例达血液学缓解的患者中，24例已停用环磷酰胺，中位随访时间39.0（8.0～56.0）个月，环磷酰胺停药后中位TFR期未达到，预估12个月TFR率为（90.87±6.16）％，36个月TFR率为（75.72±11.04）％；初治组与复发/难治组环磷酰胺的TFR差异无统计学意义（*P*＝0.451）。

**结论:**

环磷酰胺单药口服可以有效治疗T-LGLL，且患者停药后有望维持长期TFR。

## Abstract (en)

**Objective:**

To evaluate the efficacy of cyclophosphamide in patients with T-cell large granular lymphocytic leukemia（T-LGLL）and the maintenance of treatment-free remission（TFR）following drug discontinuation.

**Methods:**

Clinical data were collected from 37 patients with T-LGLL who received oral cyclophosphamide at the Regenerative Medicine Clinic of the Institute of Hematology and Blood Diseases Hospital between June 2019 and March 2024. Patient clinical characteristics, treatment efficacy, and long-term TFR were analyzed.

**Results:**

The median age of the 37 patients was 60 years（range: 37–86）, and 22（59.5％）were male. Anemia was observed in 30 patients（81.1％）, and 28（75.7％）met the diagnostic criteria for secondary pure red cell aplasia. Neutropenia occurred in 15 patients（40.5％）, lymphocytosis in 11（29.7％）, and thrombocytopenia in three（8.1％）. Sixteen patients（43.2％）had not received prior immunosuppressive therapy（treatment-naive group）, while 21 patients（56.8％）were refractory to or had relapsed after immunosuppressive treatment（refractory/relapsed group）. All patients met the treatment criteria and received oral cyclophosphamide at doses of 50–100 mg/day. Among the 36 evaluable patients, hematologic remission was achieved in 25（69.4％）, with a median time of 2.0 months（range: 0.7–7.0）. There was no statistically significant difference in remission rates between the treatment-naive and refractory/relapsed groups（68.5％ *vs*. 66.7％, *P*＝0.589）. Among the 25 patients who achieved hematologic remission, 24 discontinued cyclophosphamide. With a median follow-up of 39.0 months（range: 8.0–56.0）, the median TFR duration was not reached. The estimated TFR rates were（90.87± 6.16）％ at 12 months and（75.72±11.04）％ at 36 months. No significant difference in TFR was observed between the treatment-naive and refractory/relapsed groups（*P*＝0.451）.

**Conclusion:**

Oral cyclophosphamide is effective in the treatment of T-LGLL, and patients may maintain long-term TFR following drug discontinuation.

T细胞大颗粒淋巴细胞白血病（T-LGLL）是一类以细胞毒性T淋巴细胞克隆扩增为主要特征的惰性淋巴增殖性疾病。环磷酰胺、甲氨蝶呤、环孢素是一线免疫抑制剂，其中环磷酰胺往往有较好的治疗反应，整体有效率为53％～78％[1]–[2]。但由于T-LGLL发病率低、临床表现隐匿、易被漏诊的特点，国内针对环磷酰胺单药在T-LGLL中的应用并不常见，停药后的长期随访数据不足。本研究回顾性分析了T-LGLL患者单用环磷酰胺后的疗效及停药后的长期无治疗缓解（TFR）情况，以提高对本病诊疗的认识。

## 病例与方法

1. 病例：纳入2019年6月至2024年3月于中国医学科学院血液病医院确诊的37例T-LGLL患者，应用流式细胞术免疫分型、T细胞受体（TCR）Vβ检测、TCR基因重排等方法检测大颗粒淋巴细胞（LGL）及其克隆表达情况，所有患者均符合T-LGLL诊断标准[3]–[5]：①相应的临床症状、体征、血细胞减少等；②外周血LGL持续增多；③LGL表达特征性免疫表型；④克隆性LGL。继发性纯红细胞再生障碍（PRCA）诊断标准[6]：①HGB低于正常值（男性<120 g/L，女性<110 g/L）；②网织红细胞百分比<1％，网织红细胞绝对值<10×10^9^/L；③骨髓红细胞系统各阶段显著低于正常值，有核红细胞比例<5％，粒系及巨核系的各阶段在正常范围内。本研究经中国医学科学院血液病医院伦理委员会批准（批件号：QTJC2025014-EC-1）。

2. 治疗方案：所有患者明确诊断及治疗指征后均接受环磷酰胺口服治疗，具体剂量为100 mg/d；2例患者治疗过程中因不良反应将环磷酰胺减量为50 mg/d（1例出现3级血小板减少，1例出现2级ALT/AST升高）；治疗至少4个月后进行疗效评价，如有效，则建议患者继续口服环磷酰胺，6个月内停药。

3. 疗效标准：至少治疗4个月后根据外周血细胞计数评价治疗效果。结合患者的基线症状以及治疗后血象变化，血液学治疗反应定义如下[1]–[4]，完全血液学缓解（HCR）：血细胞计数恢复至同年龄段正常水平，且LGL计数在正常范围内；血液学部分缓解（HPR）：血细胞计数增长，但未恢复至完全正常，中性粒细胞绝对计数>0.5×10^9^/L但<1.5×10^9^/L，或输血需求减少但仍然是输血依赖状态；治疗无效（NR）：患者治疗4个月后仍未见上述治疗反应。血液学缓解包括HCR与HPR。

4. 随访：通过查阅门诊/住院病历及电话/微信随访方式获取患者疗效情况，随访截止日期为2024年7月30日，中位随访时间为28（2～61）个月。TFR期定义为环磷酰胺治疗有效的患者停药至重新开始治疗或死亡或随访截止的时间。

5. 统计学处理：采用SPSS23.0和R4.1.2软件进行数据分析，计数资料用例数（百分比）表示，计量资料用*M*（范围）或均值±标准差表示。定性资料采用独立样本*t*检验和Fisher检验，分组比较采用Mann-Whitney *U*检验和Kruskal-Wallis检验。TFR分析采用Kaplan-Meier分析方法，*P*<0.05为差异有统计学意义。

## 结果

1. T-LGLL患者治疗前基线：37例患者中男22例（59.5％），女15例（40.5％）；中位年龄60（37～86）岁。患者均因自觉不适就诊，未伴有B症状，13例（35.1％）患者用药前伴有脾大。所有患者治疗前均存在外周血细胞计数异常，30例（81.1％）表现为贫血，28例（75.7％）符合继发性PRCA标准，15例（40.5％）粒细胞减少/缺乏，11例（29.7％）淋巴细胞计数增多，3例（8.1％）血小板减少（图1）。35例（94.6％）促红细胞生成素水平升高，30例（81.1％）血清铁蛋白水平升高，9例（24.3％）LDH升高。

**图1:**
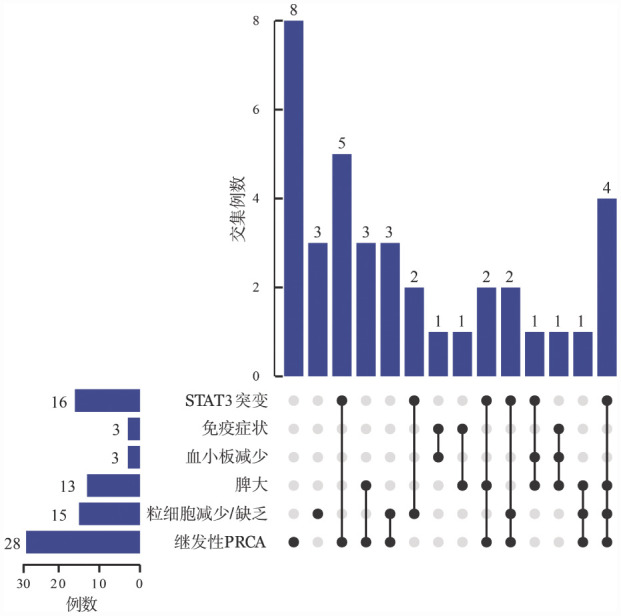
37例T细胞大颗粒淋巴细胞白血病患者临床特征分布情况 **注** PRCA：纯红细胞再生障碍

26例（70.3％）LGL计数>0.5×10^9^/L；LGL克隆性检测显示35例（94.6％）患者TCR重排阳性；33例患者接受TCRVβ克隆性检测，其中31例（93.9％）检测到单克隆T淋巴细胞。16例（43.2％）STAT3突变。31例（83.8％）治疗前CD3^+^ T细胞比例异常增高，28例（75.7％）CD3^+^CD8^+^ T细胞比例异常增高。

16例患者既往未接受任何免疫抑制治疗（初治组）、21例接受环孢素或甲氨蝶呤或糖皮质激素治疗无效或治疗后复发（复发/难治组）。两组患者接受环磷酰胺治疗前的HGB、中性粒细胞绝对计数、PLT、淋巴细胞及LGL计数差异均无统计学意义（均*P*>0.05，表1）。但复发/难治组的初始CD8^+^ T细胞（2.46％对0.81％，*P*＝0.037）及中央记忆CD8^+^ T细胞（1.28％对0.49％，*P*＝0.025）比例明显高于初治组。

**表1:** 37例T细胞大颗粒淋巴细胞白血病患者临床特征

特征	总体（37例）	初治组（16例）	复发/难治组（21例）	统计量^a^	*P*值^a^
性别（例，男/女）	22/15	9/7	13/8	0.120	0.631
年龄［岁，*M*（范围）］	60（37～86）	67（43～86）	57（37～83）	2.032	0.050
HGB［g/L，*M*（范围）］	67（35～137）	74（35～134）	66（46～137）	0.389	0.700
ARC［×10^9^/L，*M*（范围）］	20.7（0～240.0）	30.2（0～139.0）	11.8（0～240.0）	0.128	0.899
ANC［×10^9^/L，*M*（范围）］	1.04（0.01～11.45）	0.80（0.01～4.57）	1.47（0.39～11.45）	−1.911	0.064
PLT［×10^9^/L，*M*（范围）］	204（27～484）	167（74～399）	311（27～484）	−1.918	0.051
LY［×10^9^/L，*M*（范围）］	2.01（0.20～8.25）	2.10（0.20～6.20）	1.99（0.45～8.25）	0.359	0.722
LGL计数［×10^9^/L，*M*（范围）］	1.27（0.02～4.86）	1.38（0.02～4.86）	1.16（0.10～4.25）	1.275	0.211
CD3^+^ T细胞比例［％，*M*（范围）］	95.2（68.7～98.9）	95.0（68.7～98.0）	95.4（68.7～98.9）	0.270	0.789
CD3^+^CD8^+^ T细胞比例［％，*M*（范围）］	65.6（25.0～91.7）	75.0（25.0～89.0）	59.0（26.0～91.7）	0.965	0.342
初始CD8^+^ T细胞比例［％，*M*（范围）］	1.74（0.16～27.13）	0.81（0.26～5.98）	2.46（0.16～27.13）	−2.231	0.037
中央记忆CD8^+^ T细胞比例［％，*M*（范围）］	0.83（0.02～4.04）	0.49（0.04～2.17）	1.28（0.02～4.04）	−2.356	0.025
效应CD8^+^ T细胞比例［％，*M*（范围）］	55.16（1.82～89.66）	68.92（3.28～87.39）	53.85（1.82～89.66）	0.877	0.387
TCR重排阳性［例（％）］	35（94.6）	16（100）	19（90.5）	1.611	0.495
TCRVβ单克隆（检测出单克隆的例数/接受检测的例数）	31/33	13/14	18/19	0.050	0.676

**注** ^a^初治组与复发/难治组比较；ARC：网织红细胞绝对计数；ANC：中性粒细胞绝对计数；LY：淋巴细胞绝对值；LGL：大颗粒淋巴细胞；TCR：T细胞受体

2. 环磷酰胺疗效及安全性：37例患者中1例用药1.5个月后因出现急性脑梗死停药，可评价疗效的36例患者中达血液学缓解25例（69.4％），中位达血液学缓解时间为2.0（0.7～7.0）个月。19例（52.8％）达HCR，中位达HCR时间为5.0（2.0～18.0）个月。中位随访时间28.0（2.0～61.0）个月，36例患者存活，1例死亡。该例死亡患者接受环磷酰胺100 mg/d治疗2周，因2级肝功能损伤环磷酰胺减量为50 mg/d，治疗1个月达HPR，治疗时长总计9个月；停药3个月后突发头痛，影像学检查提示中枢神经系统脑膜瘤可能，并出现全身多处骨骼异常信号影，血象持续为缓解状态，骨髓活检可见异常细胞、推测为转移瘤伴骨髓纤维化，保守治疗效果不佳，患者1个月后死亡。

11例初治患者（11/16，68.5％）接受环磷酰胺治疗后达血液学缓解，中位达血液学缓解时间为2.5（1.0～4.0）个月；8例（8/16，50.0％）达HCR，中位达HCR时间为5.0（2.0～7.0）个月。14例复发/难治患者（14/21，66.7％）接受环磷酰胺治疗后达血液学缓解，中位达血液学缓解时间为1.7（0.7～7.0）个月；12例（12/21，57.1％）达HCR，中位达HCR时间为4.5（2.5～10.0）个月。两组患者血液学缓解率（*P*＝0.589）及HCR率（*P*＝0.460）差异均无统计学意义。

环磷酰胺治疗期间18例（48.6％）患者出现药物相关不良反应（DAE），12例（32.4％）患者出现血液系统DAE：3例为3级粒细胞减少，1例为3级血小板减少，其余均为1～2级DAE；非血液系统DAE均为1～2级：3例患者出现恶心及纳差症状，2例患者出现肝功能异常，1例患者出现脑血管缺血，1例患者并发湿疹，1例患者出现皮肤疼痛。上述DAE在环磷酰胺减量或对症处理后均得到改善（表2）。

**表2:** T细胞大颗粒淋巴细胞白血病患者的药物相关不良事件分析

药物相关不良事件	例数	用药调整	结局
粒细胞减少			
1级	1	无	恢复正常
2级	1	无	恢复正常
3级	3	短疗程联合粒细胞刺激因子	恢复正常
血小板减少			
1级	4	无	恢复正常
2级	3	无	恢复正常
3级	1	环磷酰胺减至50 mg/d，联合海曲泊帕15 mg/d	恢复正常
血红蛋白减低（1级）	2	无	恢复正常
恶心/纳差（1级）	3	无	改善
湿疹（1级）	1	无	改善
皮肤疼痛（1级）	1	无	改善
ALT/AST升高			
1级	1	联合保肝药物	恢复正常
2级	1	暂停环磷酰胺1周，后减量为50 mg/d，并联合保肝药物	恢复正常
神经系统血栓	1	停用环磷酰胺	改善

3. 环磷酰胺停药后长期TFR：截至末次随访，25例达血液学缓解的患者中，24例已停用环磷酰胺，中位随访时间39.0（8.0～56.0）个月，接受环磷酰胺治疗的中位时间为8.0（4.0～10.0）个月，环磷酰胺停药后中位TFR期未达到，预估12个月TFR率为（90.87±6.16）％、24个月TFR率为（90.87±6.16）％、36个月TFR率为（75.72±11.04）％。

停药患者包括10例初治T-LGLL、14例复发/难治T-LGLL，初治组与复发/难治组环磷酰胺的TFR差异无统计学意义（*P*＝0.451）。预估初治组12个月TFR率、24个月TFR率、36个月TFR率分别为（88.89±10.47）％、（88.89±10.47）％、（66.67±20.78）％；复发/难治组12个月TFR率、24个月TFR率、36个月TFR率分别为（92.86±6.88）％、（92.86±6.88）％、（81.25±12.41）％。

## 讨论

T-LGLL作为少见的细胞毒性T细胞克隆扩增的惰性淋巴增殖性疾病，其一线治疗药物为环磷酰胺、环孢素与甲氨蝶呤，3种免疫抑制剂的选择取决于患者诊断时的临床特征，既往文献报道有效率为30％～85％[7]–[11]。鉴于高达40％患者贫血症状突出，国内多数诊疗中心以环孢素作为首选免疫抑制剂，但患者往往需要长期环孢素维持治疗[7]–[8]。系列回顾性研究发现小剂量环磷酰胺的有效率为53％～78％，本中心数据显示环磷酰胺有效率为69.4％，与国际水平基本一致，且与环孢素整体有效率（48％～74％）接近[4],[11]。此外，本中心接受环磷酰胺治疗患者的中位血液学缓解时间为2个月，与文献报道中环孢素的中位起效时间接近（8周）[7]–[8]，进一步提示口服小剂量环磷酰胺对于T-LGLL具有快速起效及高有效率的特点。

目前认为环磷酰胺、环孢素与甲氨蝶呤3种免疫抑制剂可以作为T-LGLL患者一线及二线治疗选择，对于既往接受过环孢素、甲氨蝶呤治疗的患者，即使为无效或复发状态，依然可以通过口服小剂量环磷酰胺达到理想的疗效[2]–[4],[12]–[14]。本中心的数据显示，既往免疫抑制治疗失败的T-LGLL患者启动环磷酰胺治疗后有效率达66.7％，与初治T-LGLL患者的有效率差异无统计学意义（66.7％对68.5％，*P*＝0.589）。这也进一步证实了3种免疫抑制剂之间无交叉耐药[8]–[9],[11]–[13]。

为尽可能降低环磷酰胺的累积毒性影响，建议T-LGLL应用环磷酰胺的时间不超过1年，环磷酰胺停药后患者疗效是否能维持是困扰临床医师的关键问题。法国一项纳入229例患者的回顾性临床研究队列发现24例接受环磷酰胺治疗的T-LGLL患者无进展生存时间为31（12～60）个月[13]，本中心数据进一步明确中国患者环磷酰胺停药后TFR情况，25例有效患者中，24例已停用环磷酰胺，中位随访时间39.0（8.0～56.0）个月，中位TFR期未达到，预计12个月TFR率为（90.87±6.16）％、24个月TFR率为（90.87±6.16）％、36个月TFR率为（75.72±11.04）％。上述数据提示环磷酰胺有望为T-LGLL患者带来长期TFR，减少疾病负担。

本研究通过对接受环磷酰胺治疗的T-LGLL患者诊疗经过的分析及长期随访，发现环磷酰胺在T-LGLL中具有较好的疗效，并且有望为患者带来长期TFR，可以优先考虑其作为一线治疗选择。由于本研究为单中心回顾性研究，样本量小，未来有待更大样本的前瞻性研究进一步验证。
